# Disability and loneliness in the United Kingdom: cross-sectional and longitudinal analyses of trends and transitions

**DOI:** 10.1186/s12889-023-17481-y

**Published:** 2023-12-19

**Authors:** Eric Emerson, Roger J. Stancliffe, Zoe Aitken, Jodie Bailie, Glenda M. Bishop, Hannah Badland, Gwynnyth Llewellyn, Anne M. Kavanagh

**Affiliations:** 1 Centre for Disability Research, Faculty of Health and Medicine, Lancaster University, Lancaster, UK; 2 Centre for Disability Research and Policy, Faculty of Medicine and Health, University of Sydney, Camperdown, NSW 2006 Australia; 3 College of Nursing and Health Sciences, Flinders University, Bedford Park, SA 5042 Australia; 4 Melbourne School of Population and Global Health, The University of Melbourne, Melbourne, VIC 3010 Australia; 5 University Centre for Rural Health, University of Sydney, Camperdown, NSW 2006 Australia; 6 School of Public Health, University of Sydney, Camperdown, NSW 2006 Australia; 7 Social and Global Studies Centre, RMIT University, Melbourne, VIC 3001 Australia

**Keywords:** Disability, Loneliness, Inequality, Transitions, Trajectories

## Abstract

**Background:**

Loneliness can have a detrimental impact on health, yet little is known about the association between disability and loneliness.

**Methods:**

Secondary analysis of three waves of data collected between 2017 and 2020 by the UK’s annual household panel study, *Understanding Society*. Direct age-standardisation was used to compare the prevalence of loneliness at each wave and the persistence of loneliness across all three waves for participants with/without disabilities aged 16–65 years. Transitional probabilities for the stability of loneliness, the stability of non-loneliness, the onset of loneliness and the offset of loneliness between consecutive waves were also estimated.

**Results:**

At each wave, the prevalence of loneliness was significantly higher among respondents with disabilities than respondents without disabilities; these inequalities persisted with no evidence of change over time. The prevalence of persistent loneliness was 46% for respondents with disabilities compared with 22% for respondents without disabilities. Risk factors for the likelihood of persistent loneliness included disability, financial stress, not living as a couple, living in rented accommodation, being female and not being employed. The probability of the onset and stability of loneliness between successive waves were markedly higher for people with disabilities compared with people without disabilities.

**Conclusion:**

Adults with disabilities were more likely to experience loneliness, become lonely and remain lonely over time than their peers. Policies and interventions aimed at reducing loneliness should ensure that they are accessible and effective for people with disabilities. Further research is needed to explore the health outcomes of persistent loneliness among people with/without disabilities.

**Supplementary Information:**

The online version contains supplementary material available at 10.1186/s12889-023-17481-y.

## Introduction

The degree to which individuals are interconnected and embedded in communities has a powerful impact on mortality, physical and mental health [1]. Knowledge in this area is based on a range of approaches for conceptualising and measuring social connections, including social network analysis, measuring levels of social support, social isolation and, more recently, the experience of loneliness [1]. Loneliness has been defined primarily as an emotional state; ‘*a subjective unpleasant or distressing feeling of a lack of connection to other people, along with a desire for more, or more satisfying, social relationships.’* [2] Systematic reviews suggest that loneliness is associated with future mortality, [3] physical health, [4] and mental health [5]. A small number of studies (primarily undertaken with older adults) suggest that, in contrast to intermittent or transient loneliness, persistent loneliness is associated with poorer health outcomes, including mortality and indicators of physical (e.g., cardiovascular health) and mental health, including severe mental illness and dementia [6–11]. There is some evidence that the prevalence of loneliness may be increasing over time (at least among younger adults) and may have shown an increase following the onset of the COVID-19 pandemic [12, 13].

Known risk factors for experiencing loneliness include female gender, younger age, not being married/partnered, experiencing partner loss, living alone, being unemployed, having limited social networks, low level of social activity, poor self-reported health or mental health difficulties [13–15]. Risk factors for the onset or persistence of loneliness include not being married or living as a couple, high exposure to adverse childhood experiences, poor health or mental health, lower educational attainment or cognitive functioning, and higher socio-economic stress [16–18].

There is also growing evidence that people with disability are more likely to report being lonely, [19–27] as well as having fewer friends, less social support, and being more socially isolated than those without disability [28, 29]. Risk factors for experiencing loneliness among people with disabilities include longer-term persistent disability, younger age, low socio-economic position, not being employed, living in rented accommodation, low level of education, not being married, living alone, living in urban areas, lower levels of internet use, lack of transport, poorer self-rated health and having a mental health-related disability [21, 22, 26, 30, 31].

Two notable weaknesses in the current loneliness literature are the paucity of evidence on: (1) trends over time in loneliness among people with disabilities); and (2) transitions into and out of loneliness using longitudinal data in general, and for people with disabilities [32]. Our aims respond to these knowledge gaps by:

1. Estimating the extent of, and trends over time, in disability-related inequalities in the experience of loneliness in the United Kingdom (UK) among adults aged 16–65 years;
2. Estimating whether there are differences between people with and without disability in terms of onset and persistence of loneliness;
3. Identifying risk factors for the persistence of loneliness over time among adults with disabilities.

## Method

We undertook secondary analysis of data collected in *Understanding Society*, the UK’s annual household panel study (https://www.understandingsociety.ac.uk/). *Understanding Society* is an initiative funded by the Economic and Social Research Council and various UK Government Departments, with scientific leadership by the Institute for Social and Economic Research, University of Essex, and survey delivery by NatCen Social Research and Kantar Public. The research data are distributed by the UK Data Service. Full details of the survey’s development and methodology are available in a series of publications, [33, 34] key aspects of which are summarised below.

### Sampling and procedure

In the first wave of data collection (2009–2011), random sampling from the Postcode Address File in Great Britain and from the Land and Property Services Agency list of domestic properties in Northern Ireland identified 55,684 eligible UK households. New individuals enter the survey if they: (a) are living in a participating household and attain the age of 16 years; or (b) become resident in a participating household. Individuals leave the survey if they: (a) no longer give consent to participate; (b) cannot be traced; or (c) move outside the UK. Participants are notified of the timing of their interview by mail and email and are regularly contacted between Waves with information about the results of the surveys [35].

Data collection for each wave of *Understanding Society* takes place primarily over a two-year period. This study uses data collected between 2017 and 2020. At Wave 9 (2017-18), full interviews were completed with 24,564 individuals aged 16–65 years, the target population for the present study. At Wave 10 (2018-19), full interviews were completed with 26,666 individuals aged 16–65 years. At Wave 11 (2019-20), full interviews were completed with 25,177 individuals aged 16–65 years. Estimated individual response rates were constant at approximately 80% for Waves 9–11. The total unweighted sample size across all three Waves was 31,616.

Data collection for variables used in the present paper was undertaken using a combination of computer-assisted personal interviewing and computer-assisted self-completion. Median interview lengths at Wave 9 were approximately 12 min for the Household Questionnaire and 42 min for the Individual Questionnaire [35].

### Measures

#### Disability

Disability was ascertained by an affirmative response to two questions. First, ‘Do you have any long-standing physical or mental impairment, illness or disability? By ‘long-standing’ I mean anything that has troubled you over a period of at least 12 months or that is likely to trouble you over a period of at least 12 months.’

Second, if respondents gave an affirmative response to the first question, they were asked ‘Does this/Do these health problem(s) or disability(ies) mean that you have substantial difficulties with any of the following areas of your life?’. The response options (all that applied were coded) were: (1) mobility; (2) lifting, carrying or moving objects; (3) manual dexterity; (4) continence; (5) hearing (apart from using a standard hearing aid); (6) sight (apart from wearing standard glasses); (7) communication or speech problems; (8) memory or ability to concentrate, learn or understand; (9) recognising when you are in physical danger; (10) physical co-ordination; (11) difficulties with own personal care; (12) other. These categories are based on the UK’s Government Statistical Service’s harmonised impairment standard (https://analysisfunction.civilservice.gov.uk/policy-store/impairment/). Respondents who reported difficulties in one or more of these areas of functioning were counted as having disability in that wave of the survey. Disability data were missing for 0.0-0.4% of participants across waves.

#### Loneliness

Loneliness was measured by the UK’s Government Statistical Service harmonised measure of loneliness. This includes four separate items which were first introduced into *Understanding Society* in W9. Items 1–3 were taken from a short form of the UCLA Loneliness Scale [36]. The fourth item has recently been included in UK surveys as a headline indicator for the measurement of loneliness [37].

1. *How Often Do You Feel That You Lack Companionship?*
2. *How often do you feel left out?*
3. *How often do you feel isolated from others?*
4. *How often do you feel lonely?*

Response options for all items were: hardly ever or never, some of the time, often. The four items evidenced strong internal consistency (Cronbach’s alpha = 0.90), and all loaded strongly on the first extracted component of an unrotated principal components analysis which accounted for 76.5% of variation. Factor scores were recoded to generate a simple ordinal scale with a distribution similar to sample responses to individual items; no/low loneliness (60%), moderate loneliness (30%), substantial loneliness (10%) [20]. We also created a binary measure of loneliness at each wave from this variable (substantial/moderate loneliness vs. no/low loneliness). In addition, we undertook sensitivity analyses for the fourth item only given it is included in other UK surveys as the national headline indicator for the measurement of loneliness [37]. Loneliness data were missing for 4.3-6.0% of participants.

To examine onset of loneliness, for pairs of successive waves (W9/W10 and W10/11) we calculated transition probabilities for all four possible options using the binary measure of substantial/moderate loneliness: no/low loneliness in Wn, no/low loneliness in Wn + 1 (stable no/low loneliness); no/low loneliness in Wn, substantial/moderate loneliness in Wn + 1 (loneliness onset); substantial/moderate loneliness in Wn, no/low loneliness in Wn + 1 (loneliness offset); and substantial/moderate loneliness in Wn, substantial/moderate loneliness in Wn + 1 (stable loneliness). Transition probabilities were calculated as the proportion of respondents in each state at Wn who remained in that state at Wn + 1 or transitioned to a different state at Wn + 1 (loneliness onset/loneliness offset).

To examine the persistence of loneliness over time we created a measure of persistent loneliness across the three waves of data collection using the binary measure of loneliness (substantial/moderate loneliness vs. no/low loneliness) creating a categorical variable describing persistent loneliness (substantial/moderate lonelinessin all three waves), intermittent loneliness (substantial/moderate loneliness in one or two waves) and never lonely (no/low loneliness in all three waves). These data were missing for 45.3% of the 31,616 respondents who participated in one or more of the three waves. Missingness was primarily related to participants not participating in all three waves of data collection.

#### Covariates

##### Demographics

Information was collected on age group in ten-year bands, sex and ethnicity (White UK/Other ethnicity). Age and sex data were available for all respondents. Ethnicity data were missing for 0.4% of participants.

##### Living arrangements and socio-economic position

We derived a binary indicator of current living arrangements from the available data (living as a couple with another adult in the household vs. other living arrangements; data missing for < 0.1% of participants). We also derived three binary indicators of socio-economic position (SEP): (1) self-reported current financial strain (comfortably off/doing all right/just about getting by vs. finding things difficult/very difficult; data missing for 2.0-2.9% of participants); (2) living in rented accommodation vs. other options (data missing for 2.5-2.9% of participants); and (3) employed/fulltime student vs. unemployed/not in the labour force (data missing for 0.1-0.2% of participants).

##### Urban/rural location

*Understanding Society* data are released with a binary indicator of urban/rural location based on the household address falling within an urban settlement with a population of 10,000 or more as defined by the Office for National Statistics Rural and Urban Classification of Output Areas 2001. These data were missing for 0.1-1.9% of participants.

### Approach to analysis

Unweighted sample sizes were 24,564 (4,431 with disability) for Wave 9, 26,666 (4,803 with disability) for Wave 10 and 25,177 (4,703 with disability) for Wave 11. A summary of the sample characteristics disaggregated by disability status across for Waves 9 is provided in Table 1.

**Table 1:** Sample characteristics (weighted) with 95% confidence intervals for Wave 9

Total n (unweighted)	24,564	
	Disability	No disability
Disability n (unweighted)	4,431	20,133
Age		
16–29	14.2% (12.8–15.9)	25.5% (24.6–26.5)
30–49	33.4% (31.5–35.3)	41.5% (40.4–42.5)
50–65	52.4% (50.4–54.4)	33.1% (32.2–33.9)
Sex		
Male	42.8% (41.0-44.6)	48.9% (48.2–49.7)
Female	57.2% (55.4–59.0)	51.1% (50.3–51.8)
Ethnicity		
White UK	89.1% (87.8–90.2)	85.7% (84.9–86.5)
Other	10.9% (9.8–12.2)	14.3% (13.5–15.1)
Living arrangements		
Living as a couple	52.3% (50.3–54.3)	61.2% (60.1–62.2)
Not	47.7% (45.7–49.8)	38.8% (37.9–39.9)
Financial strain		
Yes	17.5% (16.0-19.2)	6.9% (6.3–7.5)
No	82.5% (80.8–84.0)	93.1% (92.5–93.7)
Living in rented accommodation		
Yes	51.2% (48.9–53.6)	30.7% (29.5–32.0)
No	48.8% (46.5–51.1)	69.3% (68.0-70.5)
Employed or full-time student		
Yes	51.2% (49.2–53.1)	84.4% (83.6–85.0)
No	48.8% (46.9–50.8)	15.7% (15.0-16.4)
Location		
Urban	79.0% (77.0-80.8)	77.2% (76.0-78.4)
Rural	21.0% (19.2–23.0)	22.8% (21.6–24.0)
Loneliness		
No/low	38.7% (36.8–40.6)	62.6% (61.7–63.6)
Moderate	37.3% (35.4–39.3)	29.2% (28.3–30.1)
Substantial	24.0% (22.3–25.7)	8.2% (7.6–8.7)

#### Loneliness

We generated direct age standardised estimates of the prevalence of no/low, substantial and moderate loneliness among adults with/without disabilities for each of Waves 9–11. Standardisation was made to the age distribution (in 5-year age bands) of respondents with disabilities in the weighted Wave 11 sample. As such, the prevalence estimates for loneliness remain accurate for adults with disabilities, with estimates for adults without disabilities being adjusted to match the age profile of adults with disabilities [38]. In addition, we used Poisson regression to estimate prevalence rate ratios (adjusted for age, sex and ethnicity) for substantial and moderate loneliness (with no/low loneliness the reference group) for respondents with disabilities compared with those without disability for each wave and explored possible interactions between disability status and age group and sex.

#### Persistent loneliness

We generated direct age standardised estimates (as above, to the age distribution of respondents with disabilities) of the prevalence of no/low loneliness, intermittent and persistent loneliness across the three waves. In addition, we used Poisson regression to identify predictors of persistent loneliness across all three waves of data collection. Covariates included in the model were age (in years), sex, ethnicity, living as a couple or not, living in rented accommodation, employment status, self-assessed financial situation and urban location. These covariates were selected for inclusion in the Poisson model to minimise confounding bias and generate valid estimates of the association between disability and persistent loneliness [39, 40].

#### Loneliness dynamics

We estimated transitional probabilities for the stability of loneliness, stability of non-loneliness, the onset of loneliness and the offset of loneliness between consecutive waves (W9-10, W10-11) using the binary measure of loneliness (substantial/moderate loneliness vs. no/low loneliness).

Given the modest amount of missing data in most analyses, complete case analyses were undertaken. For analyses adjusted for sex, age and ethnicity total missingness varied between 3.4% in Wave 11 to 6.2% in Wave 10. Analyses that included all covariates had a total missingness of 8.9%. However, our measure of the persistence of loneliness over three waves involved significant amounts of missing data. Given the high level of missingness, we undertook a sensitivity analysis in which we conducted multiple imputation by chained equations to create 25 parallel datasets in which loneliness and covariate data were fully imputed. Analyses were conducted in each imputed dataset and results were summarised across the datasets using Rubin Rules.

All analyses were undertaken in Stata 16 using the Survey Data Analysis routines to address clustering in the sample design and inverse probability weights provided by the data owners to address biases in initial recruitment and attrition over time. The mi commands were used for the multiple imputation.

## Results

The prevalence of disability ranged from 19.4% (95% CI 18.7–20.2) in Wave 9 to 20.7% (95% CI 20.0-21.5) in Wave 11. People with disabilities were more likely than their peers to be older, female, majority ethnic status, not to be living as a couple, report financial strain, live in rented accommodation, not be employed or a full-time student, and live in urban locations (Table 1). There was minimal variation in sample characteristics across waves.

### Trends in the prevalence of loneliness over time

**Fig. 1:**
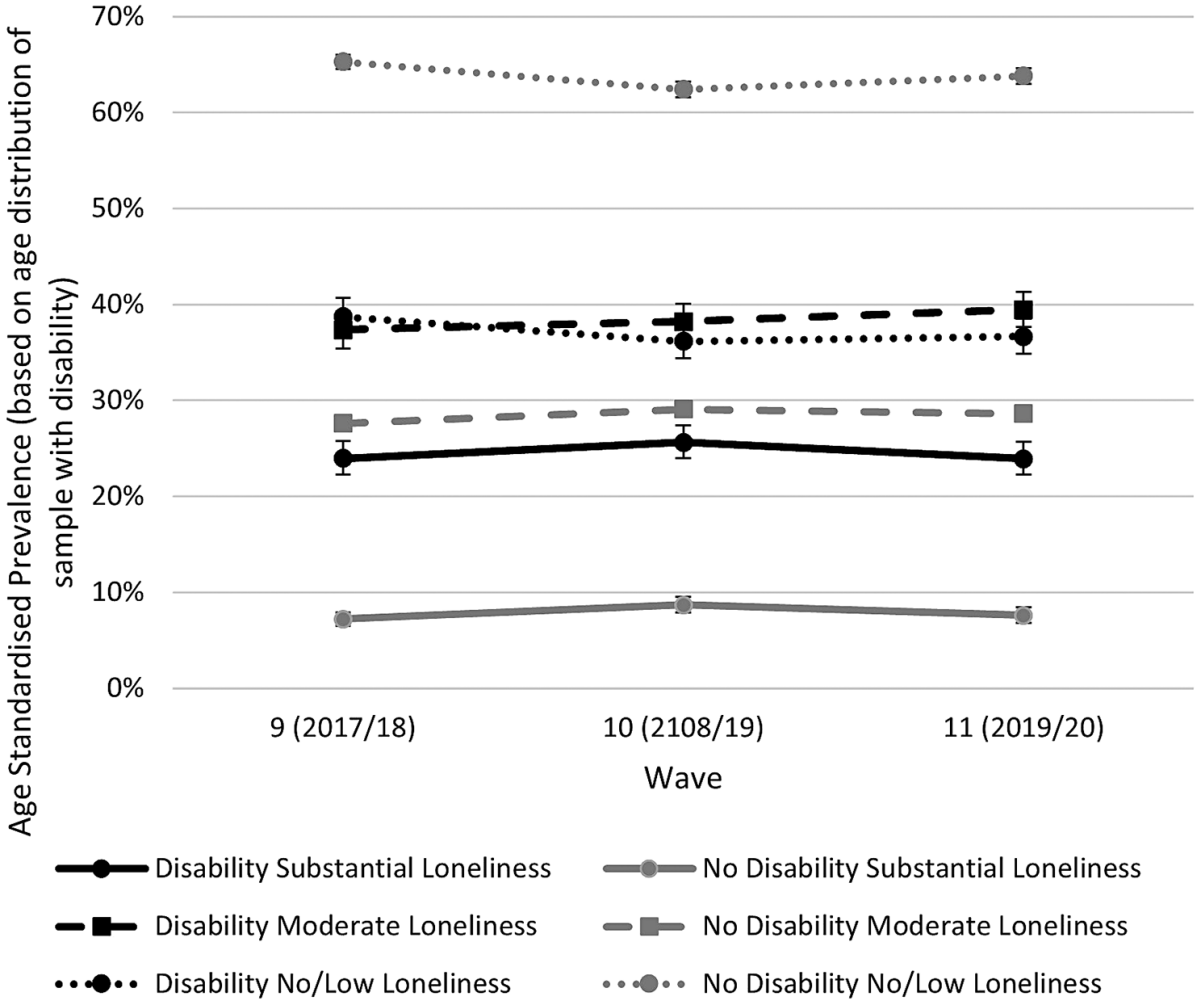
Age standardised estimates of the prevalence of no/low, moderate and substantial loneliness among adults with/without disabilities for Waves 9–11 (2017 2020)

Age standardised estimates of the prevalence of no/low, substantial and moderate loneliness among adults with/without disabilities for Wave 9 (2017–2019), Wave 10 (2018–2020) and Wave 11 (2019–2021) are presented in Fig. 1. For each wave the prevalence of both substantial and moderate loneliness was significantly higher among respondents with disabilities than among respondents without disabilities. For example, in Wave 9, the prevalence of substantial loneliness for people with disabilities was 24.0% compared with 7.2% for people without disabilities. There was little evidence of changes in prevalence of loneliness or the magnitude of the differences in loneliness between people with and without disabilities across waves. Similar results were obtained using the single-item national headline indicator for loneliness (Supplementary Fig. 1).

The Poisson models demonstrated that the prevalence of substantial loneliness was 3.78 times greater (95% CI 3.45–4.14, *p* < 0.001) for people with disabilities compared with those without disabilities for Wave 9, 3.20 (2.94–3.49, *p* < 0.001) for Wave 10 and 3.54 (3.24–3.87, *p* < 0.001) for Wave 11, respectively, adjusted for age, sex and ethnicity. The prevalence of moderate loneliness was 1.65 times greater in Wave 9 (95% CI 1.56–1.74, *p* < 0.001), 1.60 (1.52–1.68, *p* < 0.001) for Wave 10 and 1.66 (1.57–1.75, *p* < 0.001) for Wave 11 for people with disabilities compared with those without disabilities respectively, adjusted for age, sex and ethnicity.

Analyses addressing the association between disability and loneliness indicated that significant disabilityXsex and disabilityXage-groupXsex interactions occurred at two of the three waves. As a result, the above analyses were repeated stratified for broad age group and respondent sex. Results are presented in Supplementary Table 1. Inspection of the results highlighted two consistent patterns: (1) the highest disability-related relative risk of both moderate and substantial loneliness for both men and women occurred in the in 50–65 years age group; and (2) disability-related relative risk of substantial loneliness was higher for men than women at each wave and age group. However, while the patterns are consistent, there were overlapping of confidence intervals between estimates.

### Persistent loneliness

The estimated age-standardised prevalence of no/low, intermittent loneliness and persistent loneliness across the three waves of data collection for respondents with disabilities was 19.6% (18.4–20.7), 34.1% (32.4–35.8) and 46.4% (44.4–48.4). Corresponding estimates for respondents without disabilities were 42.2% (41.5–43.0), 35.6% (34.8–36.3) and 22.2% (21.6–22.8). The sensitivity analysis gave very similar results (with disabilities: 20.2% (19.0-21.4), 34.4% (32.9–36.1) and 45.3% (43.5–47.2); without disabilities: 42.3% (41.5–43.2), 35.6% (34.9–36.3) and 21.8% (21.3–22.4)). The likelihood of persistent loneliness is over three times greater for adults with disabilities (PRR = 3.37) (Table 2) when controlling for between group differences in a range of possible covariates including age, sex, ethnicity, relationship status, three indicators of socio-economic position and urban/rural location.

**Table 2:** Association between disability, covariates and prevalence rate ratio (95% confidence limits) of persistent and intermittent loneliness (reference group = no/low loneliness for all three waves)

Complete case analysis	Intermittent loneliness	Persistent Loneliness
Disability	1.79*** (1.56–2.05)	3.37*** (2.93–3.86)
Age (in years)	0.98*** (0.98–0.99)	0.98*** (0.98–0.98)
Female (ref group = male)	1.27*** (1.16–1.39)	1.35*** (1.16–1.39)
Minority ethnicity (ref group = White UK)	1.14 (0.98–1.32	0.90 (0.74–1.10)
Not living as a couple (ref group = not)	1.49*** (1.35–1.67)	2.50*** (2.17–2.70)
Living in rented accommodation (ref group = not)	1.35*** (1.20–1.52)	1.42*** (1.25–1.61)
Not employed or full-time student (ref group = not)	1.08 (0.95–1.22)	1.27** (1.10–1.47)
Financial situation difficult/very difficult (ref group = not)	2.09*** (1.66–2.63)	2.92*** (2.26–3.77)
Urban location (ref group = rural)	1.06 (0.95–1.17)	1.08 (0.95–1.23)
Multiple imputation by chained equations	Intermittent loneliness	Persistent Loneliness
Disability	1.76*** (1.57–1.98)	3.26*** (2.90–3.68)
Age (in years)	0.98*** (0.98–0.99)	0.98*** (0.98–0.98)
Female (ref group = male)	1.26*** (1.17–1.36)	1.40*** (1.28–1.51)
Minority ethnicity (ref group = White UK)	1.07 (0.94–1.19)	0.89 (0.77–1.02)
Not living as a couple (ref group = not)	1.49*** (1.37–1.64)	2.38*** (2.13–2.63)
Living in rented accommodation (ref group = not)	1.29*** (1.16–1.43)	1.36*** (1.22–1.52)
Not employed or full-time student (ref group = not)	1.14* (1.02–1.27)	1.33*** (1.18–1.49)
Financial situation difficult/very difficult (ref group = not)	2.02*** (1.67–2.44)	2.99*** (2.42–3.68)
Urban location (ref group = rural)	1.04 (0.95–1.15)	1.07 (0.96–1.20)

Note: * p < 0.05, ** p < 0.01, *** p < 0.001

### Loneliness dynamics

Loneliness transitional probabilities are presented in Table 3 for respondents with/without disabilities separately for transitions between Waves 9–10 and Waves 10–11. The probability of onset and persistence of loneliness was higher for people with disabilities compared with people without disabilities (e.g., in Waves 9–10, onset: 0.29 (i.e., 29% of respondents with disabilities who were not lonely in Wave 9 were lonely in Wave 10) versus 0.21; persistence: 0.82 versus 0.74) and probabilities of offset and persistence of no/low loneliness were lower (e.g., in Waves 9–10, offset: 0.18 versus 0.26; persistence: 0.71 versus 0.79). For all transition probabilities, the confidence intervals for the estimates for people with and without disability did not overlap.

**Table 3:** Association between disability and the onset, offset of loneliness and the persistence of loneliness and non-loneliness in consecutive waves

	Transitional probability (95% CI)	
	With disability	No disability
*Onset of loneliness (no/low loneliness at Wave n, but lonely at Wave n + 1)*		
Waves 9–10	0.29 (0.26–0.32)	0.21 (0.20–0.22)
Waves 10–11	0.26 (0.23–0.29)	0.18 (0.17–0.19)
*Offset of loneliness (lonely at Wave n, but no/low loneliness at Wave n + 1)*		
Waves 9–10	0.18 (0.16–0.20)	0.26 (0.24–0.27)
Waves 10–11	0.20 (0.18–0.22)	0.30 (0.29–0.32)
*Persistence of loneliness (lonely at Wave n and at Wave n + 1)*		
Waves 9–10	0.82 (0.80–0.84)	0.74 (0.73–0.76)
Waves 10–11	0.80 (0.78–0.82)	0.70 (0.68–0.72)
*Persistence of no/low loneliness (no/low loneliness at Wave n and at Wave n + 1)*		
Waves 9–10	0.71 (0.68–0.74)	0.79 (0.78–0.80)
Waves 10–11	0.74 (0.71–0.77)	0.82 (0.81–0.83)

## Discussion

### Main findings of this study

Secondary analysis of data collected in three successive waves of *Understanding Society* (the UK’s annual national household panel survey) indicated that at each wave the prevalence of both substantial and moderate loneliness was significantly higher among respondents with disabilities than among respondents without disabilities. For example, in Wave 9, the prevalence of substantial loneliness for people with disabilities was 24.0% compared with 7.2% for people without disabilities. These inequalities were persistent with little evidence of changes in prevalence of loneliness or the magnitude of the differences in loneliness between people with and without disabilities across the three waves. The estimated age-standardised prevalence of persistent loneliness across the three waves of data collection was 46.4% for respondents with disabilities compared with 22.2% for respondents without disabilities. The probability of onset and persistence of loneliness between successive waves were markedly higher for people with disabilities compared with people without disabilities.

### What is already known on this topic

There is growing evidence that people with disabilities are more likely to report being lonely than their non-disabled peers [19–27]. Reported risk factors for experiencing loneliness among people with disabilities include younger age, low socio-economic position, not being employed, living in rented accommodation, low level of education, not being married, living alone, living in urban areas, lower levels of internet use, lack of transport, poorer self-rated health, having a mental health-related disability and with low levels of access to environmental assets [19–22, 26, 30, 31].

### What this study adds

This is, to our knowledge, the first published study that has investigated the association between disability and the persistence of loneliness and transitions into/out of loneliness. Our finding that people with disability are twice as likely as their non-disabled peers to be exposed to persistent loneliness is important given that evidence, albeit mainly based on studies of older adults, suggests that persistent loneliness is associated with poorer health outcomes than intermittent or transient loneliness [8–11]. In addition, identification of risk factors for the likelihood of persistent loneliness (under financial stress, not living as a couple, living in rented accommodation, being female and not being employed or a full-time student) identify subpopulations of people with disabilities that are particularly at risk and draws attention to potential pathways for developing preventative interventions.

### Implications for practice

Public health interventions addressing socially determined health inequity, including the health impacts associated with loneliness, need to take account of the situation of marginalised or vulnerable groups [41]. People with disability are increasingly being recognised as one such group [42]. Approximately 49% of UK adults who experience persistent loneliness will have disabilities. It is critically important therefore that all policies and interventions aimed at reducing the levels of loneliness in the population ensure that they make reasonable adjustments/accommodations so that these policies/practices are accessible and effective for people with disabilities. Making such adjustments/accommodations is also a requirement for countries that have signed the UN Convention on the Rights of Persons with Disabilities and, in the UK, under the Equalities Act 2010. Examples of potentially important adjustments/accommodations include ensuring that: (1) all settings in which interventions take place are fully accessible to people with restricted mobility, limited access to transport, receptive and expressive communication impairments or sensory differences as a result of their disability; (2) all intervention materials (e.g., advertising of interventions, self-help guides) are fully accessible to people with sensory or intellectual impairments; (3) all processes of interventions are fully accessible to people with restricted mobility, people with cognitive impairments, people with sensory/social differences and people with restricted stamina and provide a sense of psychological safety for people with diverse impairments. Ensuring that appropriate adjustments/accommodations are made could be facilitated by working with people with disabilities and Disabled People’s Organisations to audit existing and proposed interventions.

### Limitations of this study

The main strengths of the study are the use of the Office for National Statistics harmonised question set to identify people with disabilities and the use of a large-scale nationally representative longitudinal survey data. The main limitations of the study are: (1) the omission from the sampling frame of people not living in ordinary households which would include people with disabilities living in institutional settings including group homes; (2) lack of reasonable adjustments in the survey which is likely to have excluded some adults with more severe disabilities; and (3) the large amount of missing data due to sample attrition. However, regarding the latter point it is important to note that full imputation of missing data produced very similar results to complete case analysis.

### Future research

Further research is needed to explore the health and wellbeing outcomes of persistent loneliness among people with disabilities and the extent to which these may vary across types of functional impairments associated with disability, such as intellectual disability.[e.g., 43, 44] Finally, the data presented were collected in the period leading up to and including the early stages of the COVID-19 pandemic. Future data releases will enable researchers to determine whether similar loneliness patterns exist following the COVID-19 pandemic.

### Electronic supplementary material

Below is the link to the electronic supplementary material.

Supplementary Material 1

## Data Availability

The data used are available conditional on approval free of charge to researchers through the UK Data Service (https://ukdataservice.ac.uk/).

## Abbreviations

UK: United Kingdom
W: Wave
CI: confidence interval

## Declarations

### Authors’ information

Within the research team, two researchers (GB, AK) identified as having disability, and three researchers (GL, AK, JB) identified as having a family member with disability. All authors are deeply committed to reducing the inequalities experienced by people with disability.
