# Supplementary material for: Disability and loneliness in the United Kingdom: cross-sectional and longitudinal analyses of trends and transitions
Source: BMC Public Health. 2023 Dec 19;23:2537. doi: 10.1186/s12889-023-17481-y (PMC10729364; doi:10.1186/s12889-023-17481-y)
Supplement: Supplementary file 1 — Supplementary Material 1 [file 12889_2023_17481_MOESM1_ESM.docx]

# Supplementary Materials

| Supplementary Table 1: Prevalence rate ratios adjusted for ethnicity and age (in years) for the association between disability and substantial/moderate loneliness (respondents without disability being the reference group) | | | | |
| --- | --- | --- | --- | --- |
|  | Loneliness | Wave 9 | Wave 10 | Wave11 |
| Age 16-29 |  |  |  |  |
| Men | Substantial | 3.02*** (2.26-4.04) | 3.00*** (2.45-3.68) | 3.81*** (3.04-4.78) |
|  | Moderate | 1.51*** (1.20-1.90) | 1.56*** (1.30-1.89) | 1.78*** (1.52-2.08) |
| Women | Substantial | 2.44*** (1.93-3.09) | 2.29*** (1.88-2.79) | 2.13*** (1.73-2.63) |
|  | Moderate | 1.59*** (1.36-1.85) | 1.26** (1.06-1.49) | 1.51*** (1.31-1.73) |
| Age 30-49 |  |  |  |  |
| Men | Substantial | 4.72*** (3.66-6.08) | 3.03*** (2.40-3.83) | 3.83*** (3.01-4.86) |
|  | Moderate | 1.61*** (1.37-1.88) | 1.63*** (1.42-1.87) | 1.59*** (1.36-1.86) |
| Women | Substantial | 3.94*** (3.31-4.70) | 3.02*** (2.57-3.54) | 3.20*** (2.65-3.87) |
|  | Moderate | 1.59*** (1.43-1.77) | 1.54*** (1.40-1.70) | 1.49*** (1.34-1.66) |
| Age 50-65 |  |  |  |  |
| Men | Substantial | 5.00*** (3.89-6.41) | 4.50*** (3.51-5.77) | 4.76*** (3.66-6.19) |
|  | Moderate | 1.85*** (1.62-2.11) | 1.87*** (1.65-2.10) | 1.93*** (1.70-2.19) |
| Women | Substantial | 3.97*** (3.28-4.79) | 3.81*** (3.21-4.52) | 4.16*** (3.46-5.01) |
|  | Moderate | 1.67*** (1.50-1.86) | 1.63*** (1.48-1.80) | 1.69*** (1.53-1.87) |
| Note: * p<0.05, ** p<0.01, *** p<0.001 | | | | |

#### Supplementary Figure 1: Age standardised estimates of the prevalence of never/hardly ever lonely, sometimes lonely and often lonely among adults with/without disabilities for Waves 9-11 (June 2017 -May 2021)
